# Dietary Mulberry leaf 1-deoxynijirimycin supplementation shortens villus height and improves intestinal barrier in fattening rabbits

**DOI:** 10.5713/ab.24.0109

**Published:** 2024-08-22

**Authors:** Shaocong Li, Tao Li, Zijie Jiang, Wenyu Hou, Qirui Hou, Boris Ramos Serrano, Adileidys Ruiz Barcenas, Yuhua Wang, Weiguo Zhao

**Affiliations:** 1 Jiangsu Key Laboratory of Sericultural Biology and Biotechnology, School of Biotechnology, Jiangsu University of Science and Technology, Zhenjiang 212100, China; 2 Plant Protein and Bionatural Products Research Center, Havana, 999075 Cuba

**Keywords:** Digestion Ability, 1-Deoxynijirimycin (DNJ), Gut Health, Microbiome, Mulberry Leaves, Rabbits

## Abstract

**Objective:**

The current study investigated the effects of mulberry 1-deoxynijirimycin (DNJ) on the digestion ability, intestinal morphology, and intestinal barrier of rabbits.

**Methods:**

A total of 36 New Zealand White rabbits (male) about 45 days old (mean body weight of 1.05±0.04 kg) were reared and commercial diets were employed, and afterwards divided into three groups (n = 12) with different levels of DNJ extract additive in feed: T_0_ (0 g/kg), T_1_ (0.35 g/kg), T_2_ (0.7 g/kg) for 28 d.

**Results:**

The results demonstrated that T_2_ decreased the average daily gain (p<0.05). T_1_ and T_2_ decreased villus height and inflammatory factor levels as compared with T_0_ (p<0.05). DNJ significantly decreased the content of valeric acid (p<0.05). The content of acetic acid, propionic acid, iso butyric acid, iso valeric acid in T_1_ were higher than those in T_0_ and T_2_ (p<0.05). The content of butyric acid in T_2_ was lower than it in T_0_ and T_1_ (p<0.05). The content of caproic acid was firstly improved then reduced as the DNJ concentration improved (p<0.05). T_2_ significantly increased the abundance of *dgA-11_gut_group* and *Christensenellaceae_R-7_group* while decreased *Bacteroide* and *Ralstonia* as compared with T_0_ (p<0.05). Compared with T_0_, T_1_, and T_2_ significantly improved the gene expression of *JAM2*, *JAM3*, *mucin4*, *mucin6* (p<0.05), T_1_ significantly decreased the expression of *occluding* while T_2_ significantly increased (p<0.05), T_2_ significantly increased the expression of *claudin1* and *claudin2* (p<0.05).

**Conclusion:**

DNJ at high level changed microbiome compositions, inhibited inflammation, and improved intestinal barrier while it decreased the growth performance and shorted villus height in rabbit jejunum by regulating short chain fatty acid compositions in rabbits.

## INTRODUCTION

The need for protein sources in animal feed has been steadily increasing in recent decades [1]. Soybean meal and fish meal are the main sources of protein supplementation in feed, however, the contradiction of soybean usage in animal feed with human food security and the limited supply of fish meal brings competition for protein supplementation for animal feed [1,2]. Therefore, the exploration of new protein sources for animal feed becomes necessary. Mulberry leaf has a long history in eastern Asian of been used as animal feed and is considered as a potential feed source, particularly as a protein source for its high protein content [3]. In addition to this, feeding mulberry leaves also can improve production of livestock in various ways such as improvement of nutrient digestion, growth performance and meat quality [4,5]. Yet, there are also reports about the negative effects of mulberry leaves feeding, like decrease in weight gain [6] while the underlying mechanism hasn’t been fully understood.

1-Deoxynijirimycin (DNJ) is an alkaloid that is highly enriched in mulberry leaf. It inhibits the activities of multiple carbohydrases like α-amylase, suppresses the mRNA and protein expression of glucose transporters [7,8]. It also possesses anti-inflammatory properties, which could prevent gastric ulcer (GU) in GU model mice and protect gastric function [9,10]. There are also reports of its positive effects on gas dysbiosis [11,12]. However, there are also negative reports of mulberry leaf, like reducing on villus height of jejunum and α-diversity indices of cecal microbiome in geese, and was identified as a factor that affects the digestion system and decreases growth [13]. Meanwhile, the exploration of the effects of DNJ on digestion system in monogastric animals is still unclear. Taking consideration the large amount of research on of some the anti-nutrition factors like tannin existing in many plants, we selected DNJ from mulberry as the subject for evaluation of its effects on animal digestion system, and tried to establish a new approach to explore the mechanism of mulberry feeding’s negative effects on livestock.

In this study, different levels of DNJ supplementation were added into feed to investigate the effects of DNJ on growth performance, digestive function, intestinal morphology and intestinal barrier in fattening rabbits. The results of this study may elucidate our understanding of DNJ from mulberry leaves as a supplement in animal feed which will be crucial to the livestock industry.

## MATERIALS AND METHODS

### Animal care

The animal experiments were approved by the Institution Animal Care and Use Committee of Jiangsu University of Science and Technology (Zhenjiang, China, No. GQ20211108).

### Animals

Thirty-six male weaned New Zealand White rabbits (45 days of old) from Anhui Xianhong Biotechnology Co., Ltd. (Suzhou, Anhui, China) with similar weight (1.05±0.04 kg) were randomly divided into three groups (T_0_, T_1_, T_2_), basic feed was supplemented with 0, 0.35, 0.7 g/kg mulberry DNJ extracts (DNJ-E, purity 40%) which were brought from Shengqing Biotechnology Co. Ltd., Xi’an, China. In a previous experiment, we found that mulberry leaves caused negative effects on growth performance in rabbits when its content in feed was ≥20%, according to Zhang et al [14], we chose 0.14 mg/g content of DNJ in mulberry leaves as standard, providing 0.35 g and 0.7 g DNJ extraction in feed, which equalled the content of DNJ when the mulberry leaves account for 10% and 20% in feed. The DNJ extracts were uniformly mixed with raw material powder of basic diets, then made into small particles that were suitable for rabbit feed. The rabbits were kept in individual cages (45 cm×50 cm×37 cm) which were equipped with feeder and watering bowls and provided with feed and water *ad libitum* in ambient temperature of 12°C to 15°C. The rabbits were fed twice per day at 8:30 and 17:30. The basic diet was prepared according to NRC rabbit standard [15]. and the composition and nutrient levels of the basic diet for rabbits are shown in Supplementary Table S1. The feeding trail was 28 d.

### Evaluation of growth performance

All rabbits were weighted twice a week, and at the end of the trial, the average daily weight gain (ADG) was calculated. The feed consumption of each rabbit was recorded daily for the calculation of average daily feed intake (ADFI) and future feed conversion rate (FCR).

### Sample collection

Faecal output of the six rabbits that were closest to the average weight of each group were collected at 8:00 from 24 d to 27 d in individual bags and stored at −20°C until analysis for apparent digestion, according to Pérez et al [16]. Six rabbits which were closest to the average weight of each group were humanely slaughtered as the way described by Nakyinsige et al [17] 12 h after the last feeding. Samples for morphological investigation were collected from the middle segment of the jejunum, transferred into 4% paraformaldehyde solution and after flushed by 0.9% saline. Samples for short chain fatty acid (SCFA) determination and microbiome analysis were collected from the cecal digesta, stored at −80°C after frozen in liquid nitrogen. Mucosa samples were scraped from jejunum and stored at −80°C after frozen in liquid nitrogen (after digestive removed and tissue rinsed with 0.9% NaCl) before being tested for either inflammatory cytokines including tumor necrosis factor alpha (TNF-α), interleukin-6 (IL-6), and C-reactive protein (CRP), or intestinal barrier related gene expression.

### Nutrition digestion determination

Dry matter (DM), crude protein (CP) (method 4.2.08), ether extract (EE) (method 920.85), ash (method 942.05), crude fibre (CF) (method 978.10), acid detergent fibre (ADF) (method 973.18), and ash determination were according to AOAC 1990 [18]. The method of neutral detergent fibre (NDF) determination was according to Van Soest et al [19]. Before determination, all the samples were dried at 80°C for 24 h to a constant weight, then ground and passed through a 1 mm screen.

### Morphological investigations

Samples of jejunum segment (around 1 cm in length) were kept in 4% paraformaldehyde solution for 48 h to fix. After that, different concentrations of ethanol solution (50%, 70%, 80%, 90%, and 100%) were used to dehydrate samples. Then, samples were transferred into paraffin for embedding after being soaked in xylene. Finally, hematoxylin and eosin (HE) were used for staining. Sections (around 5 mm) were cut by rotary microtome (Leica, Wetzlar, Germany) and captured photographed by light microscope (BX51; Olympus, Tokyo, Japan). The average of 10 randomly selected villus height and crypt depth per rabbit were calculated to be the average for each rabbit. The villus height and crypt depth were measured, and the villus height-to-crypt depth (Vh/Cd) ratio was calculated.

### Inflammatory cytokines measurement

The inflammatory cytokines, including TNF-α, IL-6, and CRP were measured by using ELISA kit (Angle Gene Biotechnology, Nanjing, China).

### Short chain fatty acid determination measurement

Samples of about 50 mg from cecal digesta were vortexed with 12.5 μL 2-ethylbutyric acid, then vortexed for 5 min with 0.5 mL methyl tert-butyl ether (MTBE) added, the supernatant of samples was taken after centrifuging at 1,500 g for 15 min. GC-MS analysis (2030/TQ-8040NX; Shimadzu, Japan), a flame ionization detector and a capillary column (DB-WAX; 30 m×0.32 mm×0.5 μm; Agilent Technologies, Santa Clara, CA, USA) was used. Measurement conditions were followed: the injector/detector temperature of 230°C/240°C, the gas flow rate was 1 L/min, the initial temperature maintained at 80°C for 2 min, then raised to 180°C and maintained for 3 min by 10°C/min, finally raised to 230°C and maintained for 2 min by 40°C/min.

### Microbiome analysis

Cecal digesta (0.5 g) was used for DNA extraction using DNA isolation kit (Power Soil DNA Isolation Kit; Mobio Laboratories Inc., Carlsbad, CA, USA). Afterwards the DNA was assessed with a spectrophotometer (Onedrop OD-1000+ spectrophotometer; Onedrop, Shanghai, China) and 1% agarose gel electrophoresis. The V3 to V4 hypervariable regions of the bacterial 16S rRNA gene were amplified by polymerase chain reaction (PCR) (Mastercycler nexus PCR; Eppendorf, Hamburg, Germany) with primer (F:5′-CCT AYGGGRBGCASCAG-3′, R:5′-GACTACNNGGGTATC TAAT-3′), GoTaq Hot Start GoTaq Hot Start Colorless Master Mix (Promega, Madison, WI, USA) and DNA polymerase (Promega, USA). The PCR products were evaluated by PicoGreen fluorescent real-time DNase assay (Invitrogen, Carlsbad, CA, USA) and purified by 2% agarose gel and QIAquick PCR Purification Kit (Qiagen, Hilden, Germany). The quality of final products was evaluated by PicoGreen fluorescent real-time DNase assay and Agilent 2200 Tapestation System (Agilent Technologies, USA). Illumina Miseq pair-end 300 sequencing platform was used for 16s rRNA gene sequencing.

### RNA isolation and real time quantitative polymerase chain reaction

Total RNA of mucosa samples from jejunum was isolated by RNA isolation kit (R701; Vazyme, Nanjing, China) according to the manufacturer’s instructions, after assessing the concentrations and the purity with ND 1000 Spectrophotometer (Nano-Drop Technologies, Wilmington, DE, USA), the reverse transcription kit (PC5401; Vazyme, China) was used for reverse transcription with the reaction conditions as follows: 37°C for 15 min, then 85°C for 15 s, finally cooling to 4°C. The real-time quantitative PCR (RT-qPCR) was carried out by StepOne Plus Real time PCR instrument (Agilent, USA). Thereafter, 20 μL reaction system consisted of 10 μL 2× ChamQ SYBR qPCR Master Mix, 0.4 μL each of forward and reverse primer, 2 μL cDNA and 7.2 μL distilled water was made. The PCR cycling conditions were performed at 95°C for 30 s, followed by 40 cycles of 95°C for 10 s, and 60°C for 30 s, then the dissolution curve was generated with raising temperature to 95°C. The 2^−ΔΔCT^ method was applied to the calculation of the relative expression levels of mRNA expression. *GAPDH* was elected to be the internal reference gene for the determination of intestinal barrier related genes. All primer sequences are shown in Table 1.

### Statistical analysis

After evaluation of the data distribution through SPSS 20.0 (SPSS Inc., Chicago, IL, USA) with Shapiro-Wilk test, one-way analysis of variance were applied to analyse the data sets showing normal distribution while Kruskal-Wallis test were applied to analyse the data sets not showing normal distribution. Treatment trends were statistically compared using orthogonal polynomial contrasts. The results are presented as mean±standard error of the mean and significant differences were at p<0.05. Tables were made by Microsoft Excel and Figures were constructed by GraphPad Prism 9.0 (San Diego, CA, USA). With taking SCFA as independent variable and villus height as dependent variable, regression analysis was performed after Pearson’ coefficient calculated.

## RESULTS

### 1-Deoxynijirimycin has negative effects on growth performance in rabbits

In this experiment, T_2_ decreased ADG significantly (p<0.05) as compared with T0 (Table 2), while caused no significant differences on ADFI and FCR (p>0.05).

### 1-Deoxynijirimycin regulated nutrient digestion in rabbits

The nutrient digestion of rabbits in each group was determined as presented in Table 3, While there were no significant differences on digestion of DM, CP, EE, ash, CF, NDF, and ADF among three groups, there was a trend of increasing on digestion of DM and EE (p>0.05).

### 1-Deoxynijirimycin significantly shortened villus height of jejunum in rabbits

**Figure 1:**
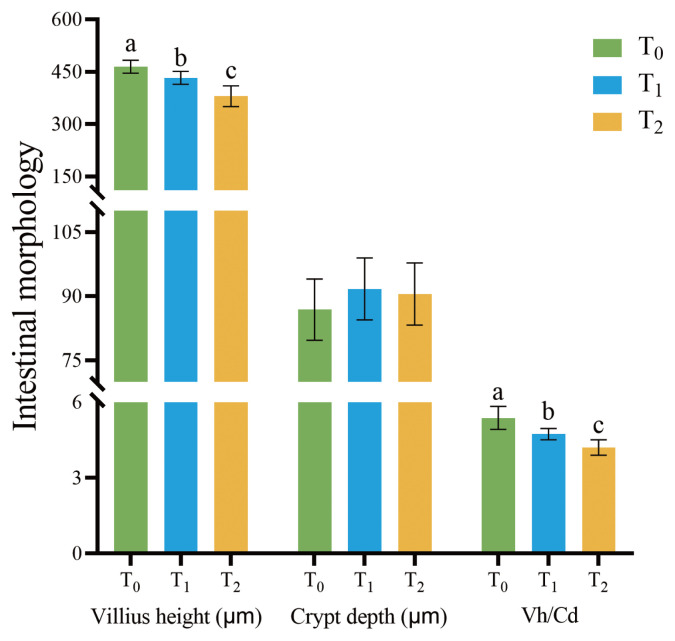
Effects of DNJ extract on the villus height and crypt depth in jejunum. DNJ, 1-deoxynijirimycin. T_0_ = 0 g/kg DNJ extract supplement; T_1_ = 0.35 g/kg DNJ extract supplement; T_2_ = 0.7 g/kg DNJ extract supplement. ^a–c^ No letter or the same letter on the top of bars mean the difference is not significant at the level of p>0.05, marking different lowercase letters on the top of bars mean the difference is significant at the level of p<0.05.

**Figure 2:**
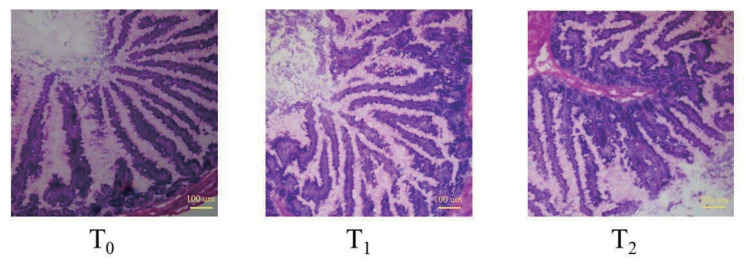
The morphology of jejunum segments by hematoxylin and eosin staining (40× microscope). DNJ, 1-deoxynijirimycin. T_0_ = 0 g/kg DNJ extract supplement; T_1_ = 0.35 g/kg DNJ extract supplement; T_2_ = 0.7 g/kg DNJ extract supplement.

Further, the jejunum morphology was investigated. As presented in Figure 1 and 2, the villus height in T_1_ and T_2_ was significantly shorter compared with T_0_ (p<0.05), but there was no significant difference in crypt depth among the groups (p>0.05), so that there was a significant decreasing of the Vh/Cd ratio in T_0_ (p<0.05).

### 1-Deoxynijirimycin diminished inflammatory cytokines in rabbit jejunum

**Figure 3:**
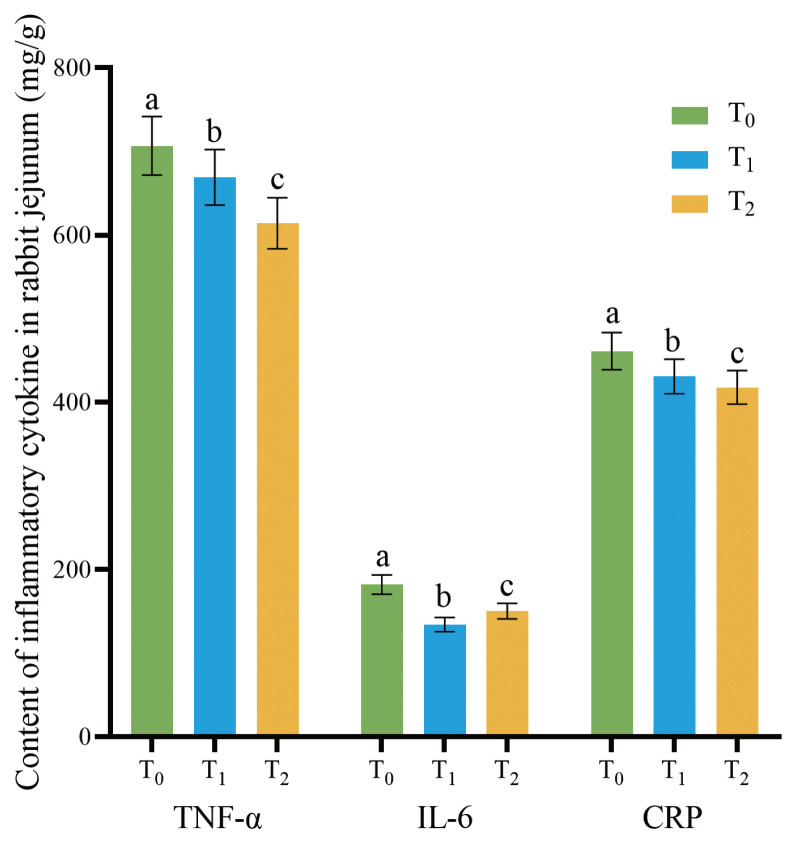
Effects of DNJ extract on inflammatory cytokines content in rabbit jejunum. DNJ, 1-deoxynijirimycin. T_0_ = 0 g/kg DNJ extract supplement; T_1_ = 0.35 g/kg DNJ extract supplement; T_2_ = 0.7 g/kg DNJ extract supplement. ^a–c^ No letter or the same letter on the top of bars mean the difference is not significant at the level of p>0.05, marking different lowercase letters on the top of bars mean the difference is significant at the level of p<0.05.

As presented in Figure 3, the levels of TNF-α, IL-6, and CRP in rabbit jejunum decreased as the DNJ content in feed increased (p<0.05).

### 1-Deoxynijirimycin changed short chain fatty acid compositions in rabbit cecum

We measured SCFA content in caecum, as presented in Table 4, DNJ significantly decreased the content of valeric acid (p<0.05). The content of acetic acid, propionic acid, iso butyric acid, iso valeric acid in T_1_ were higher than those in T_0_ and T_2_ (p<0.05). The content of butyric acid in T_2_ was lower than it in T_0_ and T_1_ (p<0.05). The content of caproic acid was firstly increased then reduced as the DNJ concentration increased (p<0.05).

### 1-Deoxynijirimycin shortened the villus height by changing short chain fatty acid compositions in rabbits

Different kinds of SCFA have different effects on intestinal epithelial cells, like butyric acid provided nutrient for cell proliferation while propionic acid suppressed it [20]. Therefor we chose the different kinds of SCFA as an independent variable and took the villus height as a dependent variable to establish a regression equation. Consideration of the number of groups, the Linear by Linear test was additionally applied to check the linear effects of linear by linear association (LLA) between the SCFA content and villus height. The results presented that butyric acid, valeric acid and caproic acid were the main SCFA that affect the villus height in rabbit intestine by DNJ, among them, the butyric acid and the valeric acid were positively correlated with the villus height while the iso valeric acid was negatively correlated with it (Table 5).

### 1-Deoxynijirimycin effected microbiome compositions

**Figure 4:**
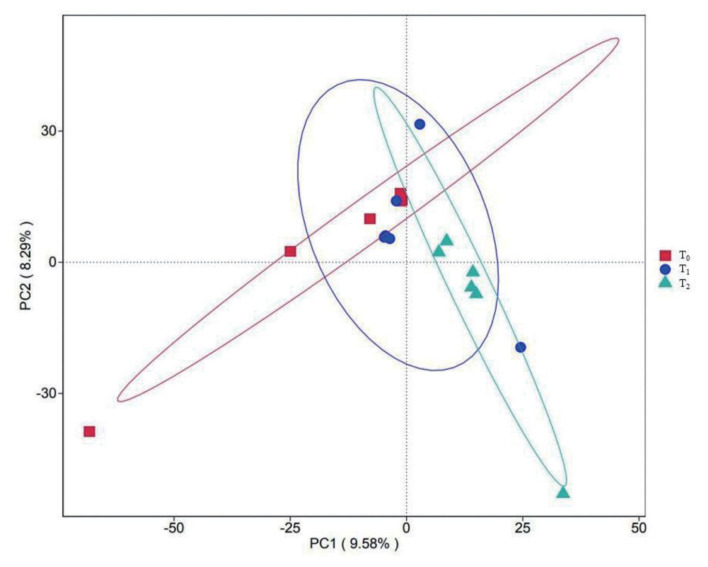
Principal component analysis (PCA) of cecal microbiome in rabbits from three groups. DNJ, 1-deoxynijirimycin. T_0_ = 0 g/kg DNJ extract supplement; T_1_ = 0.35 g/kg DNJ extract supplement; T_2_ = 0.7 g/kg DNJ extract supplement.

**Figure 5:**
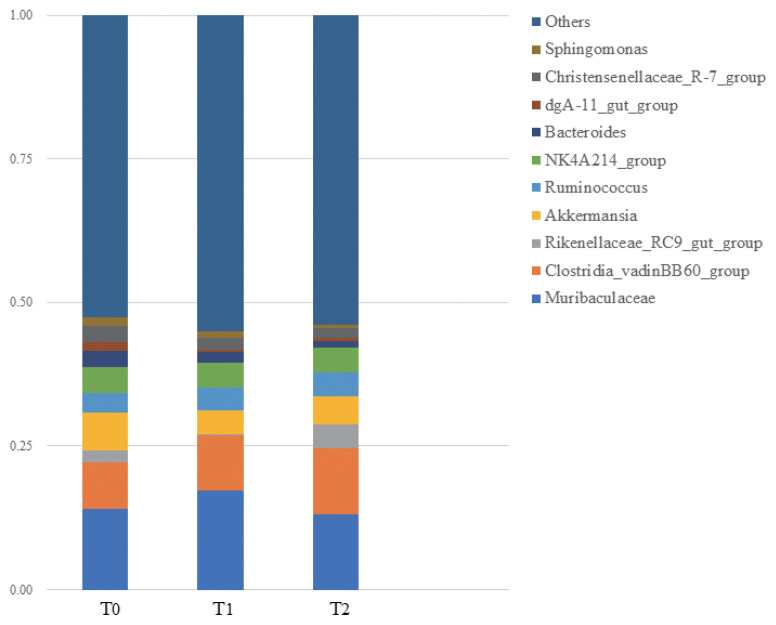
The relative abundance of several genera cecal microbiome at genus level of rabbits in the 3 groups. T0 = 0 g/kg DNJ extract supplement; T1 = 0.35 g/kg DNJ extract supplement; T2 = 0.7 g/kg DNJ extract supplement.

As presented in Figure 4, DNJ did make effects on compositions of cecal microbiome. However, there was no significant difference on α-diversity of microbiome in 3 groups (Table 6). Therefore we calculated the relative abundance of single species at genus level, the results indicated that *Muribaculaceae*, *Clostridia_vadinBB60_group* were considered as the dominant microbiome in rabbit caecum at genus level (Figure 5). As presented in Table 7, DNJ significantly improved the relative abundance of *dgA-11_gut_group* and *Christensenellaceae_R-7_group* notwithstanding the reduction of relative abundance of *Bacteroides*, *Ralstonia* (p<0.05).

### DNJ improved gene expression of intestinal barrier in rabbit jejunum

**Figure 6:**
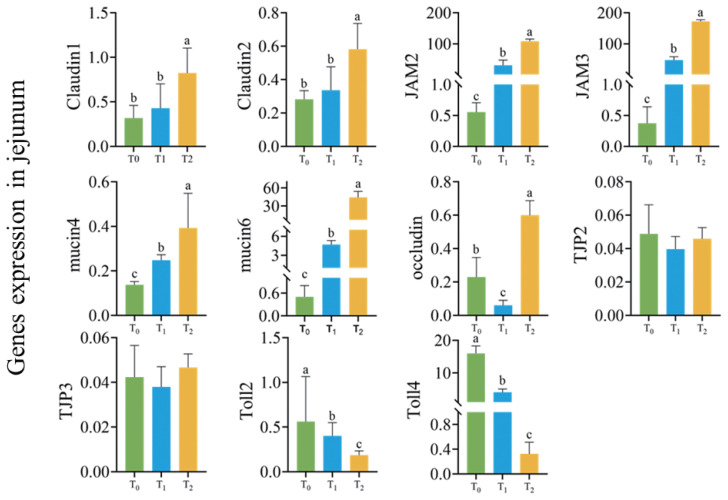
Effects of DNJ on genes expression of intestinal barrier in rabbit jejunum. DNJ, 1-deoxynijirimycin. T_0_ = 0 g/kg DNJ extract supplement; T_1_ = 0.35 g/kg DNJ extract supplement; T_2_ = 0.7 g/kg DNJ extract supplement. No letter or the same letter on the top the bar means the difference is not significant at the level of p>0.05, marking different lowercase letters means the difference is significant at the level of p<0.05.

As presented in Figure 6, DNJ significantly improved gene expression of intestinal barrier in rabbits, including *JAM2*, *JAM3*, *mucin4*, *mucin6* (p<0.05), decreased the expression of *Toll2* and *Toll4* (p<0.05), the expression of *claudin1* and *claudin2* in T_2_ significantly higher than T_0_ and T_1_ (p<0.05), the expression of occludin in T_1_ significantly lower than T_0_ and T_2_ while T_0_ significantly lower than T_2_ (p<0.05). There was no significant difference on the expression of *TJP2* and *TJP3* (p>0.05).

## DISCUSSION

Despite a long history of playing a role in animal feeding, mulberry leaves haven’t been applied in feed on a large scale. The anti-nutritional factors in mulberry leaves like digestive enzyme inhibitor might be the explanation for its limited use [21,22], while DNJ can inhibit the activity of amylase itself [23]. In this study, we evaluated the effects of DNJ on digestion ability and gut health especially intestinal health by providing feeds with different contents of DNJ. The results suggested that DNJ could affect digestion ability and gut health in rabbits and provided the theoretical basis for mulberry leaves using as feed.

Glycogen is the primary source of energy for animals. DNJ has been shown to suppress the activity of α-glucosidase, down-regulated mRNA and protein expression of sodium glucose transport protein (SGLT1), Na^+^/K^+^-ATPase and glucose transporter 2 (GLUT2) in intestines [24], leading to increased glycogen intake by animals. Kim et al [25] demonstrated that DNJ could affect the weight of high-fat-mice by regulating food intake through improving adiponectin levels in the hypothalamus. Similarly, Hou et al [26] claimed that DNJ supplementation decreased the body weight of geese but caused no significant effects on ADFI. Keeping in view these studies, a high content of DNJ could potentially affect body weight of rabbits. As to the different effects of DNJ on ADFI observed in various studies, that might be attributed to the different health conditions of animals.

Hou et al [26] found that DNJ could improve the activity of amylase, inhibit the activity of trypsin, alter microbiome compositions, and affect the nutrition digestion in geese. In this study, 0.14 to 0.28 g/kg DNJ resulted in an insignificant decrease of the apparent digestion of DM and EE. The decrease in EE digestion indicates a reduction in the energy intake of the animal while animals can accelerate protein metabolism to supply energy when there is an insufficient energy supplement [27,28]. Additionally, the reduced glycogen intake caused by DNJ may contribute to the decrease in energy supplementation. These factors may help explain how DNJ improves the apparent digestion of CP while the shorter feeding trail might explain the different results with Hou et al [26].

Song et al [29] claimed that mulberry leaves extract could improve the villus height and Vh/Cd ratio. However, Hou et al [26] proposed that the effects of DNJ on apparent digestion was due to the shorting of villus height. There have been reports about DNJ relieving inflammation of cardiac and stomach through regulating signal pathways like JAK2/STAT6 and NF-κβ [9,10], which was same as the consequences in this study. The decreasing of inflammatory factor levels suggested that mulberry DNJ was beneficial to the health of rabbit digestion system. As the consequences of the regression equation in this study suggest, the shorting of villus height in jejunum was related to the changing of SCFA compositions in rabbit caecum, leading to impacts on nutrition digestion. There are reports about the stimulation of butyric acid on growth of intestinal villi, and the relationship with intestinal cell proliferation [30,31]. SCFA may stimulate the secretion of gastrin coming from endocrine G cells which promotes the intestinal epithelial development via the gut-brain axis [32]. Additionally, a variety of metabolites were produced in the liver by the metabolism of SCFA, such as metabolites like ketone bodies and glutamate which are nutritional substrates for the growth of intestinal mucosa. The increasing content of acetate can relieve inflammatory bowel disease [33]. Sun et al [34] found that hydrolysates from mulberry leaves could increase the content of acetic acid, butyric acid and isovalerate acid in mice intestines. In this study, DNJ regulated the compositions of SCFA in caecum, the content of acetic acid, butyric acid and valeric acid increased in T_1_, which was similar with the consequences of Sun et al [34], however, the decreasing of these in T_2_, suggested that the low level of DNJ could promote intestinal health while the effects of the high level would be counterproductive.

DNJ plays a key role in the regulation of the animal micro biome. Hu et al [11] claimed that dysbacteriosis could be induced in diabetic mice by the promotion of *Lactobacillus*, *Lactobacillus Nockii* and *Bifidobacterium*, and suppression of *Ruminococcidae*, *Klebsiella pneumoniae*, *Puccinella UCG-001*, and *Bacillus S24-7* of DNJ. Zhen et al [12] claimed that DNJ could overcome gut dysbiosis and improve the abundance and diversity of gut microbiome in high fat diet-induced nonalcoholic steatohepatitis mice. DNJ increased the abundance of *dgA-11_gut_group* and *Christensenellaceae_R-7_group* while decreased the abundance of *Bacteroides* and *Ralstonia* in this study. *DgA-11_gut_group* is involved in metabolism of animo acids, energy, and lipids [35]. There are reports of the benefits from *Christensenellaceae*, including body weight increase and inflammation relief [36–38], which is in concert with ADFI decreasing and inflammatory factors reducing in this study. Meanwhile, the overgrowth of *Bacteroides* may lead to the reducing of butyric yield and Epizootic rabbit enteropathy [39,40]. *Ralstonia* is an opportunistic pathogen worldwide and resistant to many antibiotics while it is considered as a bacteria with low pathogenicity [41], reducing the risk of it causing disease in rabbits can be presumed as the abundance of it in the microbiome decreased.

Song et al [29] suggested that mulberry leaves extract could improve the mRNA expression of *ZO1*, *claudin1* and *mucin2*, and improve intestinal function. In this study, DNJ improved the expression of *JAM2* and *JAM3* in rabbit jejunum, and this suggested that DNJ might contribute to the improvement of intestinal function by mulberry leaves. Claudin, which control ion channels, is the major contributor to intestinal barrier function [42]. JAM maintains the integrity of intestinal barrier by combining other proteins into inter-celluar synapses [43]. In this study, DNJ promoted the intestinal tight junctions in rabbit jejunum by improving the mRNA expression of intestinal tight junction genes *claudin1*, *claudin2*, *JAM2*, and *JAM3*. Intestinal mucus prevents infection and inhibits inflammation by separating bacteria from intestinal epithelial cells. In this study, DNJ improved the mRNA expression of *mucin4* and *mucin6*, which suggested that the increasing secretion of intestinal mucin, the inhibition of pathogenic microorganisms and intestine content, might lead to a healthier intestine. Toll-like receptor (TLR) is a recognition receptor family, which is released when there is a cell stress or tissue damage and remits inflammation [44]. TLRs is activated and the expression of IL-1, IL-6, and IL-8 induced when bacteria stimulate cells by producing lipopolysaccharide (LPS), besides, the expression of TNF, nicotinamide adenine dinucleotide phosphate (NAPDH) oxidase family-related genes and inducible nitric oxide enzyme iNOS in intestinal organs were induced. In this study, DNJ decreased the expression of TLRs receptor, which was consistent with the results of the inflammatory cytokines decreasing.

DNJ showed many positive effects on intestinal health in this study, except it shorted the villus height, indicated its potential to be an additive, rather not only treated as an issue which affect nutrient digestion in mulberry leaves. However, we only evaluated the effects of DNJ on digestion ability and analyzed the reason for DNJ shorting villus height in intestine *in vitro* and did not carry out the experiment *in vivo*. Other potential issues that may influence the consequences of the experiment like gender and long-term feeding were not investigated either. Moreover, ways of measuring the transcriptome could be applied to animal digestion in the future that may explain the reason for shorting villus height and effects on digestion caused by DNJ. Nevertheless, this study still explored the positive effects of DNJ, which indicated it could be a potential feed additive for the improvement of gut health in animal, and provided theoretical basis for solutions to the negative effects that are caused by mulberry leaves or DNJ in animal feed, like applying them in combination with butyric acid.

## CONCLUSION

It can be summarized that mulberry 1-deoxynijirimycin inhibits the average daily gain of rabbits, decreases the villus height, reduces inflammation by decreasing tumor necrosis factor alpha, interleukin-6, and C-reactive protein content, decreases the cecal butyric acid and valeric acid content, while increases iso butyric acid and iso valeric acid content, increases the abundance of *Christensenellaceae_R-7_group* while decreases the abundance of *Bacteroides* and *Ralstonia*, increases the expression of *claudin1*, *claudin2*, *JAM2*, *JAM3*, *mucin4*, and *mucin6*, while decreases *Toll2* and *Toll4*, improves intestinal tight junction by increasing the expression of claudin1, claudin2, *JMA2* and *JAM3*, promotes mucin secretion by increasing expression of *mucin4* and *mucin6*, reduces inflammation by decreasing *Toll2* and *Toll4*. To conclude, mulberry 1-deoxynijirimycin supplementation shortens villus height by regulating short chain fatty acid compositions while improves gut health in rabbits, and could be a potential additive in feed.

## Figures and Tables

**Table 1:** Gene-specific primers used for the analysis of rabbit gene expression

Gene	Gene Bank	Primer sequences (5′-3′)	Product size (bp)
*claudin1*	NM_001089316	F: TTTGACCCCTGTCAATTCAA	106
		R: ACAAGAACAGCAAAGTAGGG	
*claudin2*	XM_008272846	F: TGTGAACCAGATTTTCTACA	129
		R: CCCAGTTTATTACATACCGA	
*TJP1*	XM_008269782	F: AGATGTTTATTTGGGCTGT	145
		R: CATATAGCTGTTTCCTCCAT	
*TJP2*	XM_017341705	F: ACAGGTTCGAGGACTG	136
		R: AACCATCCTGACAGCTC	
*JAM2*	XM_017346699	F: ATCTGGAACTCTGCAATTTA	132
		R: CCACTTATGTTGAGGTCAT	
*JAM3*	XM_008248362	F: CTCAGAAGGCTCGTCAT	113
		R: TTTACAGCTCCTATCAAGC	
*mucin4*	XM_017347029	F: GTTCCTGAGCTGTTTGTA	100
		R: TGTTGTAGTTTGTCCACTTA	
*mucin6*	XM_017340511	F: TAAAGATCTTCCTGGGGG	118
		R: CAGAGGGCATCCTGTAG	
*Toll2*	NM_001082781	F: TTCATTTGAGGACCTGTTTA	102
		R: TGACTGTCCTATAACGATAC	
*Toll4*	NM_001082732	F: TGGATTTATCCAGGTGTAA	105
		R: ATAAGCTTTGGATAGGGTTT	
*occuldin*	XM_008262318	F: CACCACACCTCTCGC	128
		R: TCTCCCAGGGAAGTCAAT	
*GAPDH*	XM_001082253	F: CTCAATGACCACTTTGTGAA	114
		R: GTTTGAGGGCTCTTACTCCT	

**Table 2:** Effects of 1-deoxynijirimycin extract on growth performance in rabbits

Item	Groups^1)^			SEM	p-value	
	
	T_0_	T_1_	T_2_		ANOVA	Linear
Initial wight	0.99±0.20	1.08±0.17	1.03±0.22	0.03	0.49	0.64
Finial wight	2.11±0.15	2.10±0.12	1.96±0.27	0.03	0.11	0.06
ADG (g/d)	40.24^a^±6.45	36.18^ab^±6.42	33.65^b^±6.51	1.15	0.05	0.01
ADFI (g/d)	140.26±27.80	125.44±28.52	117.24±37.55	5.36	0.21	0.08
FCR	3.51±0.57	3.48±0.57	3.44±0.54	0.09	0.955	0.76

DNJ, 1-deoxynijirimycin; SEM, standard error of the mean; ANOVA, analysis of variance; ADG, average daily gain; ADFI, average daily feed intake; FCR, feed conversion rate.

1) T_0_ = 0 g/kg DNJ extract supplement; T_1_ = 0.35 g/kg DNJ extract supplement; T_2_ = 0.7 g/kg DNJ extract supplement.

a,b No letter or the same letter on the shoulder of the same row of data means the difference is not significant at the level of p>0.05, marking different lowercase letters on the shoulder of the same row of data means the difference is significant at the level of p<0.05.

**Table 3:** Effects of 1-deoxynijirimycin extract on apparent digestion in rabbits

Item	Groups^1)^			SEM	p-value	
	
	T_0_	T_1_	T_2_		ANOVA	Linear
DM	61.54±4.80	61.19±5.08	60.26±5.02	1.11	0.90	0.66
CP	74.25±6.26	77.28±6.02	82.98±6.18	1.62	0.07	0.03
EE	81.18±5.74	78.54±5.57	73.63±6.85	1.55	0.13	0.04
Ash	41.69±3.23	43.18±3.19	42.15±3.34	0.74	0.72	0.81
CF	15.42±1.25	15.65±1.28	15.12±1.41	0.30	0.79	0.70
NDF	20.59±1.97	20.82±1.90	20.84±1.80	0.42	0.97	0.82
ADF	12.99±1.16	12.33±1.15	12.93±1.36	0.28	0.60	0.93

DNJ, 1-deoxynijirimycin; SEM, standard error of the mean; ANOVA, analysis of variance; DM, dry matter; CP, crude protein; EE, ether extract; CF, crude fibre; NDF, neutral detergent fibre; ADF, acid detergent fibre.

1) T_0_ = 0 g/kg DNJ extract supplement; T_1_ = 0.35 g/kg DNJ extract supplement; T_2_ = 0.7 g/kg DNJ extract supplement.

No letter or the same letter on the shoulder of the same row of data means the difference is not significant at the level of p>0.05, marking different lowercase letters on the shoulder of the same row of data means the difference is significant at the level of p<0.05.

**Table 4:** Effects of 1-deoxynijirimycin extract on cecal short chain fatty acid content in rabbit

Item	Groups^1)^			SEM	p-value	
	
	T_0_	T_1_	T_2_		ANOVA	Linear
Acetic acid	146.80^b^±19.38	176.97^a^±19.70	134.42^b^±19.77	6.14	0.01	0.29
Propionic acid	61.41^b^±7.24	74.95^a^±7.55	57.98^b^±7.41	2.42	<0.01	0.44
Iso butyric acid	62.52^b^±7.03	78.22^a^±6.84	58.50^b^±7.53	2.60	0.01	0.35
Butyric acid	191.45^a^±17.33	202.19^a^±17.39	126.97^b^±17.22	8.92	<0.01	<0.01
Iso valeric acid	15.76^b^±1.63	21.25^a^±1.62	17.08^b^±1.65	0.67	<0.01	0.18
Valeric acid	34.32^a^±1.83	30.82^b^±1.67	25.16^c^±1.96	1.00	<0.01	<0.01
Caproic acid	11.14^b^±0.86	16.79^a^±1.41	1.26^c^±0.11	1.57	<0.01	<0.01

DNJ, 1-deoxynijirimycin; SEM, standard error of the mean; ANOVA, analysis of variance.

1) T_0_ = 0 g/kg DNJ extract supplement; T_1_ = 0.35 g/kg DNJ extract supplement; T_2_ = 0.7 g/kg DNJ extract supplement.

a–c No letter or the same letter on the shoulder of the same row of data means the difference is not significant at the level of p>0.05, marking different lowercase letters on the shoulder of the same row of data means the difference is significant at the level of p<0.05.

**Table 5:** Regression analysis of short chain fatty acid and villi height

Independent variable	Dependent variable	Correlation coefficient	B	R^2^	Adj R^2^	p-value	LLA
Acetic acid	Villus height	0.073	-	-	-	-	0.762
Propionic acid		0.260	-	-	-	-	0.283
Iso butyric acid		0.309	-	-	-	-	0.203
Butyric acid		0.628*	304.831	0.394	0.356	0.005	0.010*
Iso valeric acid		−0.157	-	-	-	-	0.517
Valeric acid		0.819**	183.574	0.671	0.651	<0.001	0.001**
Caproic acid		0.641**	386.427	0.411	0.374	0.004	0.008**

B, unstandardized b coefficient; R^2^, coefficient of determination; Adj R^2^, adjusting coefficient of determination; LLA, linear by linear association.

The shoulder scale * indicates that the two variables are correlated at the p<0.05 level and ** indicates that the two variables are correlated at the p<0.01 level.

**Table 6:** The effects of 1-deoxynijirimycin on alpha diversity index of cecal microbiome in rabbits

Item	Groups			SEM	p-value	
	
	T_0_	T_1_	T_2_		ANOVA	Linear
Chao1	726.386±69.439	754.380±66.201	797.262±60.787	16.031	0.226	0.093
Dominance	0.017±0.003	0.015±0.003	0.014±0.003	0.001	0.121	0.052
Shannon	7.741±0.463	7.906±0.425	8.115±0.433	0.104	0.364	0.163
Simpson	0.983±0.016	0.986±0.008	0.986±0.012	0.003	0.869	0.619
OTUs	726.500±69.313	753.000±65.696	793.833±58.088	15.789	0.224	0.091

DNJ, 1-deoxynijirimycin; SEM, standard error of the mean; ANOVA, analysis of variance; OTUs, operational taxonomic units.

1) T_0_ = 0 g/kg DNJ extract supplement; T_1_ = 0.35 g/kg DNJ extract supplement; T_2_ = 0.7 g/kg DNJ extract supplement.

No letter or the same letter on the shoulder of the same row of data means the difference is not significant at the level of p>0.05; marking different lowercase letters means the difference is significant at the level of p<0.05.

**Table 7:** The relative abundance of part cecal microbiome at genus level in rabbits (%)

Item	Groups^1)^			SEM	p-value	
	
	T_0_	T_1_	T_2_		ANOVA	Linear
*Muribaculaceae*	14.701±2.852	17.653±2.662	13.513±2.755	0.742	0.053	0.467
*Clostridia_vadinBB60_group*	9.232±2.330	9.229±2.436	10.227±2.388	0.540	0.710	0.481
*Akkermansia*	5.604±0.72	5.810±0.767	5.385±0.730	0.169	0.620	0.616
*NK4A214_group*	4.636±0.613	4.495±0.668	4.423±0.741	0.151	0.858	0.593
*Ruminococcus*	3.744±0.843	3.768±0.990	3.718±0.727	0.191	0.995	0.958
*Bacteroides*	4.056a±0.405	2.385b±0.338	1.254c±0.374	0.291	<0.001	<0.001
*dgA-11_gut_group*	0.469b±0.052	0.538a±0.053	0.601a±0.058	0.018	0.003	0.001
*Christensenellaceae_R-7_group*	0.469b±0.049	0.536a±0.050	0.601a±0.063	0.018	0.003	0.001
*Rikenellaceae_RC9_gut_group*	0.233±0.045	0.188±0.042	0.242±0.047	0.011	0.116	0.752
*Ralstonia*	0.011a±0.001	0.004b±0.001	-^2)^		<0.001	

SEM, standard error of the mean; ANOVA, analysis of variance.

1) T_0_ = 0 g/kg DNJ extract supplement; T_1_ = 0.35 g/kg DNJ extract supplement; T_2_ = 0.7 g/kg DNJ extract supplement.

2) Indicated that the content is below the lower limit of the machine detection content. No letter or the same letter on the shoulder of the same row of data means the difference is not significant at the level of p>0.05; marking different lowercase letters means the difference is significant at the level of p<0.05.

## SUPPLEMENTARY MATERIAL

**Supplementary Table S1**. Ingredients and nutrient levels of the basal diet (air-dry basis)
