# Supplementary material for: Dietary Mulberry leaf 1-deoxynijirimycin supplementation shortens villus height and improves intestinal barrier in fattening rabbits
Source: Anim Biosci. 2024 Aug 22;37(12):2101–12. doi: 10.5713/ab.24.0109 (PMC11541019; doi:10.5713/ab.24.0109)
Supplement: Supplementary file 1 [file ab-24-0109-Supplementary-Table-S1.pdf]

Supplement 1 Ingredients and nutrient levels of the basal diet (air-dry basis)

Ingredients	Content, %	Nutrient levels[2]	Content, %
Alfalfa Grass Meal	16.09	Crude protein	17.95
Corn	21.41	Crude fiber	14.90
Soybean Meal	15.34	Neutral detergent fiber	32.79
Gluten	22.32	Acid detergent fiber	20.48
Bran	18.03	Ether extract	3.40
Soybean Oil	3.01	Ash	7.72
$CaHPO_4$	0.40	Calcium	1.06
Rock Flour	1.50	Soluble phosphorus	0.51
Salt	0.50	Lysine	0.88
L-Lysine	0.10	Methionine + Cysteine	0.68
DL-Methionine	0.30		
Premix[1]	1.00		

Note:1 The premix provided the following per kilogram of diet: Fe 30 mg，Cu 6 mg，Zn 35 mg，Mn 8 mg，Se 0.05 mg，Co 0.3 mg，I 0.4 mg，VA 6 000 IU，VD 900 IU，VE 15 IU，VK_3 1 mg,

2 Nutrient levels were analyzed values
